# Systematic testing of three Language Models reveals low language accuracy, absence of response stability, and a yes-response bias

**DOI:** 10.1073/pnas.2309583120

**Published:** 2023-12-13

**Authors:** Vittoria Dentella, Fritz Günther, Evelina Leivada

**Affiliations:** ^a^Departament d'Estudis Anglesos i Alemanys, Universitat Rovira i Virgili, Tarragona 43002, Spain; ^b^Institut für Psychologie, Humboldt-Universitat zu Berlin, Berlin 10099, Germany; ^c^Departament de Filologia Catalana, Universitat Autònoma de Barcelona, Barcelona 08193, Spain; ^d^Institució Catalana de Recerca i Estudis Avançats (ICREA), Barcelona 08010, Spain

**Keywords:** Language Models, cognitive models, bias, language

## Abstract

Humans are universally good in providing stable and accurate judgments about what forms part of their language and what not. Large Language Models (LMs) are claimed to possess human-like language abilities; hence, they are expected to emulate this behavior by providing both stable and accurate answers, when asked whether a string of words complies with or deviates from their next-word predictions. This work tests whether stability and accuracy are showcased by GPT-3/text-davinci-002, GPT-3/text-davinci-003, and ChatGPT, using a series of judgment tasks that tap on 8 linguistic phenomena: plural attraction, anaphora, center embedding, comparatives, intrusive resumption, negative polarity items, order of adjectives, and order of adverbs. For every phenomenon, 10 sentences (5 grammatical and 5 ungrammatical) are tested, each randomly repeated 10 times, totaling 800 elicited judgments per LM (total n = 2,400). Our results reveal variable above-chance accuracy in the grammatical condition, below-chance accuracy in the ungrammatical condition, a significant instability of answers across phenomena, and a yes-response bias for all the tested LMs. Furthermore, we found no evidence that repetition aids the Models to converge on a processing strategy that culminates in stable answers, either accurate or inaccurate. We demonstrate that the LMs’ performance in identifying (un)grammatical word patterns is in stark contrast to what is observed in humans (n = 80, tested on the same tasks) and argue that adopting LMs as theories of human language is not motivated at their current stage of development.

## Significance

The synthetic language generated by recent Large Language Models (LMs) strongly resembles the natural languages of humans. This resemblance has given rise to claims that LMs can serve as the basis of a theory of human language. Given the absence of transparency as to what drives the performance of LMs, the characteristics of their language competence remain vague. Through systematic testing, we demonstrate that LMs perform nearly at chance in some language judgment tasks, while revealing a stark absence of response stability and a bias toward yes-responses. Our results raise the question of how knowledge of language in LMs is engineered to have specific characteristics that are absent from human performance.

Language Models (LMs) are algorithms trained on generating probability distributions over tokens present in their input. Building on surface similarities between human and LM-generated language, a renewed interest toward LMs as theories of the human language capacity has been growing, and numerous studies have aimed at characterizing LM capabilities in different domains of grammar (1–3). The view of LMs as models of human language, however, and the subsequent characterization of their behavior through employing constructs originally conceived with human language in mind rely on one unsettled premise: LMs emulate human linguistic behavior such that their production is akin to natural language.

The role of cognitive models is to inform about a real-world target system (4): in this case, language. Observing superficial similarities between the model and the target system is interesting, but it is not enough. Unless our theories about LMs also account for the dissimilarities that mark LM performance as distinctively non-human (5), claiming that LMs have a human-like language understanding is not fully motivated. In particular, it has not yet been demonstrated that LMs possess the fundamental ability to discern possible from impossible language (6–9). If LMs truly learn language, this should in principle entail the ability to learn and apply rules of grammar consistently and reliably. If a human *h* has learned a language rule *s*, we expect *h* to apply *s* in all contexts where *s* is productive, as well as to consistently identify the violations of *s* as such. For example, we expect adult, neurotypical speakers of English to consistently produce “I ate an apple” instead of *“I eated an apple”, and to reliably recognize the latter as ill-formed, because of having acquired both the rule that governs past formation and its exceptions. Similarly, if a LM has learned *s*, it is expected to apply it consistently in all contexts that legitimately license its use.

## Grammaticality Judgments

From an experimental perspective, this consistent ability to recognize well- and ill-formed sentences as such translates into the replicability of human judgments about what forms part of our linguistic repertoire (10, 11). Though subject to context-dependent and performance factors [e.g., fatigue, temporary distraction, short-term memory limitations (12, 13)], research in linguistics has shown that such judgments in humans are robust and reliable (10, 14–16). Even when testing users of non-standard varieties that allow for a greater degree of variation, eliciting such judgments is an optimal method of establishing the limits of people’s grammars (17). Lack of standardization and official education in a language may affect people’s ability to reflect on their language (18), which is why such tasks in humans often instruct the participants to judge the well-formedness of the stimuli based on how they use language, disregarding what is prescriptively judged as the correct way to speak. Consequently, eliciting reliable judgments of grammatical well-formedness is possible even when testing speakers of moribund varieties, who may lack formal education (19). In such cases, comparisons between speakers/signers of the same community can quantify and explain any apparent “judgment unreliability” as variation, or as a conscious effort to project a specific linguistic identity, or as an indication of ongoing language change (20), without altering the fact that human judgments, once properly elicited, provide good evidence for people’s language (11, 17–19).

Do LMs provide equally reliable judgments? At present, this question lacks a clear answer. Claiming human-like formal competence in LMs (21, 22) presupposes endorsing that these models have succeeded at extracting rules of natural language grammar from their training data. If LMs have successfully learned such rules to the point that they can be described as having human-like language competence, we expect this ability to translate into grammaticality judgments that simultaneously i) are correct and ii) do not change when a linguistic stimulus is prompted repeatedly in a task. In other words, if the sentence “I ate an apple” appears 10 times in a task, humans who have acquired the relevant rules should judge it as correct consistently, without significant wavering in their judgments. Similarly, grammatically ill-formed sentences (e.g. *“Apple ate I an”) should be largely judged as ill-formed, no matter how many times they are seen.

In relation to (ii), one aspect that merits clarification concerns the use of the term “grammaticality” instead of “acceptability”. While humans make judgments of acceptability (13), LMs can only be informative about grammaticality, for they lack the embodied cognition that drives the difference between what the official grammar rule posits as grammatically correct/wrong and what a person deems acceptable based on how they use the language in different contexts, guided by self-benefiting communicative needs such as saving face or projecting a specific identity. From this distinction between grammaticality (in LMs) and acceptability (in humans) it follows that, while LM judgments are expected to be stable across all repetitions of a given stimulus, humans may occasionally vary to a limited degree in their judgments due to temporary distractions, fatigue, an internal need to emphasize specific aspects of their identity, etc. Such performance factors are absent from LMs; hence, we expect them to show stability rates that equal or even surpass those of humans. To sum up, based on what we know from human linguistic behavior, should either accuracy of judgments, stability, or both, be missing, the belief that LMs possess human-like language competence should be reconsidered, while their validity as theories of human language becomes doubtful.

To test the status of LMs as agents showing human-like language abilities, we develop and apply a series of grammaticality judgment tasks in three different LMs. The aim is to determine how LMs perform in tasks that test their ability to recognize (un)grammatical sentences. More specifically, three research questions (RQ) derive from this aim: RQ1. Is the LM performance accurate? RQ2. Is the LM performance stable? RQ3. Does the LM performance improve in terms of either accuracy or stability if a prompt is repeatedly used?

## Materials and Methods

### Sentence Material.

The employed grammaticality judgment tasks test knowledge on 8 linguistic phenomena: plural attraction (23); anaphora (24); center embedding (25); comparative sentences (26); intrusive resumption (27); negative polarity items (28); order of adjectives (29); and order of adverbs (30). These phenomena were chosen for two reasons. First, because most of them are regularly showcased in LM outputs, so they unequivocally fall within the training domain of the tested LMs. Second, they are all evaluable without context. Since there is not much systematic testing of LMs across grammatical phenomena, we opted to include in our task battery some lesser-known phenomena, such as multiple adverb ordering, to explore how LMs behave in a wider range of grammatical domains. Each phenomenon involves 10 sentences: 5 grammatical and 5 ungrammatical. *SI Appendix*, Table S1 in Supporting Information presents the task paradigm for one phenomenon and summarizes the remaining phenomena with one sample sentence per condition. The complete list of sentences alongside their sources are available at https://osf.io/7ajxr/.

With respect to the nature of the tested stimuli, all ungrammatical sentences are ungrammatical because they involve a violation of a specific rule of grammar. None of them is classified as ungrammatical due to processing reasons. Similarly, all grammatical prompts are grammatical, regardless of how easy they are to process. For example, the grammatical prompt “The ancient manuscript that the grad student who the new card catalog had confused a great deal was studying in the library was missing a page” may be long and relatively hard to parse, but it does not violate any rule of the English grammar. The ungrammatical prompt *“The trophy that the athlete who the restaurant had hired as a spokesman was stolen later” is ungrammatical because it is missing a verb (cf. “The trophy that the athlete who the restaurant had hired as a spokesman **won** was stolen later”), not because of its length or its center embedding.

### LMs.

Each grammaticality judgment was elicited using the following prompt: “Is the following sentence grammatically correct in English? insert_sample_sentence”. The elicited yes/no judgments were coded for accuracy (1 for accurate responses, 0 for inaccurate responses) and stability (1 for change from the previous judgment given to the same prompt, 0 for absence of change). As mentioned above, for each linguistic phenomenon, a total of 10 sentences (split in two conditions: 5 grammatical sentences and 5 ungrammatical sentences) was presented. Each sentence was prompted 10 times. The obtained dataset consists of 2,400 responses (i.e., 800 judgments on 80 different sentences per each LM). The prompts from all linguistic phenomena were merged in a unified pool and were then presented variably to each LM in a randomized way, using an online list randomizer.

The prompts were given to GPT-3/text-davinci-002 in November 2022 (henceforth: davinci2); to GPT-3/text-davinci-003 in January 2023 (henceforth: davinci3); and to ChatGPT in February 2023. All models were set on default interface parameters. Davinci2 and davinci3 are GPT-3 powered models (31). Davinci2 is trained with supervised fine-tuning on human-written or highly rated text samples. Building on davinci2, davinci3 is additionally trained using Reinforcement Learning from Human Feedback (RLHF). Finally, ChatGPT is fine-tuned from a model of the GPT-3.5 series at the time of testing and again trained with RLHF. The choice of testing three successive LMs of the same family aims to find whether changes in terms of the parameters and the size of training data, generally associated with improved overall performance, entail better performance also for the task at hand. Such longitudinal analyses offer insights as to which methods may contribute toward achieving better general-task performance, while across-model comparisons are likely to reveal whether more recent LMs (e.g., GPT-4, Bard) do not suffer from the limitations of their predecessors.

### Human Data.

For comparison purposes, we elicited judgments for the same sentences from human participants (n = 80; 38 F, 42 M), recruited through the crowdsourcing platform Prolific. All participants were native speakers of English, self-identified as not neurodivergent. The experiment was conducted in line with the ethical principles of the Declaration of Helsinki. It was approved by the ethics committee of the Department of Psychology at Humboldt-Universität zu Berlin (application 2020-47).

In terms of material, we used the same sentences presented to the LMs. The sentences were split for grammatical phenomenon giving rise to 8 different lists, with 100 sentences per list/phenomenon (i.e., 5 grammatical sentences and 5 ungrammatical sentences, each repeated 10 times). As attention checks, we added two additional sentences (grammatical: “The new door is red”; ungrammatical: “*Red is new door the”), each repeated 5 times. These served as control points and are not part of the analyses. Data from 14 additional participants who did not provide the correct answer to all attention-check sentences were removed from the analyses. To keep the experiment at a reasonable length in order to ensure data quality, each participant was presented with one of these 8 lists (5 male and 5 female participants per list), with all 110 sentences presented in random order.

In terms of the process, after giving informed consent to participate in the study and providing their demographic data, participants were instructed that they would be presented with 110 sentences, and that their task was to indicate whether the sentences were or not grammatically correct in English (by pressing the C key or the N key respectively). For each trial, the screen showed 1) the exact same question given to the LMs (“Is the following sentence grammatically correct in English?”), followed by 2) the sentence itself, as well as 3) an instruction at the bottom of the screen stating “Press C if it is correct, and N if it is not correct”.

The experiment was implemented using the jsPsych toolkit (32). The median completion time for the experiment was 9.22 min. Full participant data are available at https://osf.io/7ajxr/.

## Results

Results are presented separately for accuracy, stability, and their interplay. We used (Generalized) Linear Mixed Effect Models ((G)LMMs) to analyze our data. Details about the statistical analyses and the annotated code together with datasets are available at https://osf.io/7ajxr/.

### Accuracy.

#### Accuracy by model and condition.

**Fig. 1.**
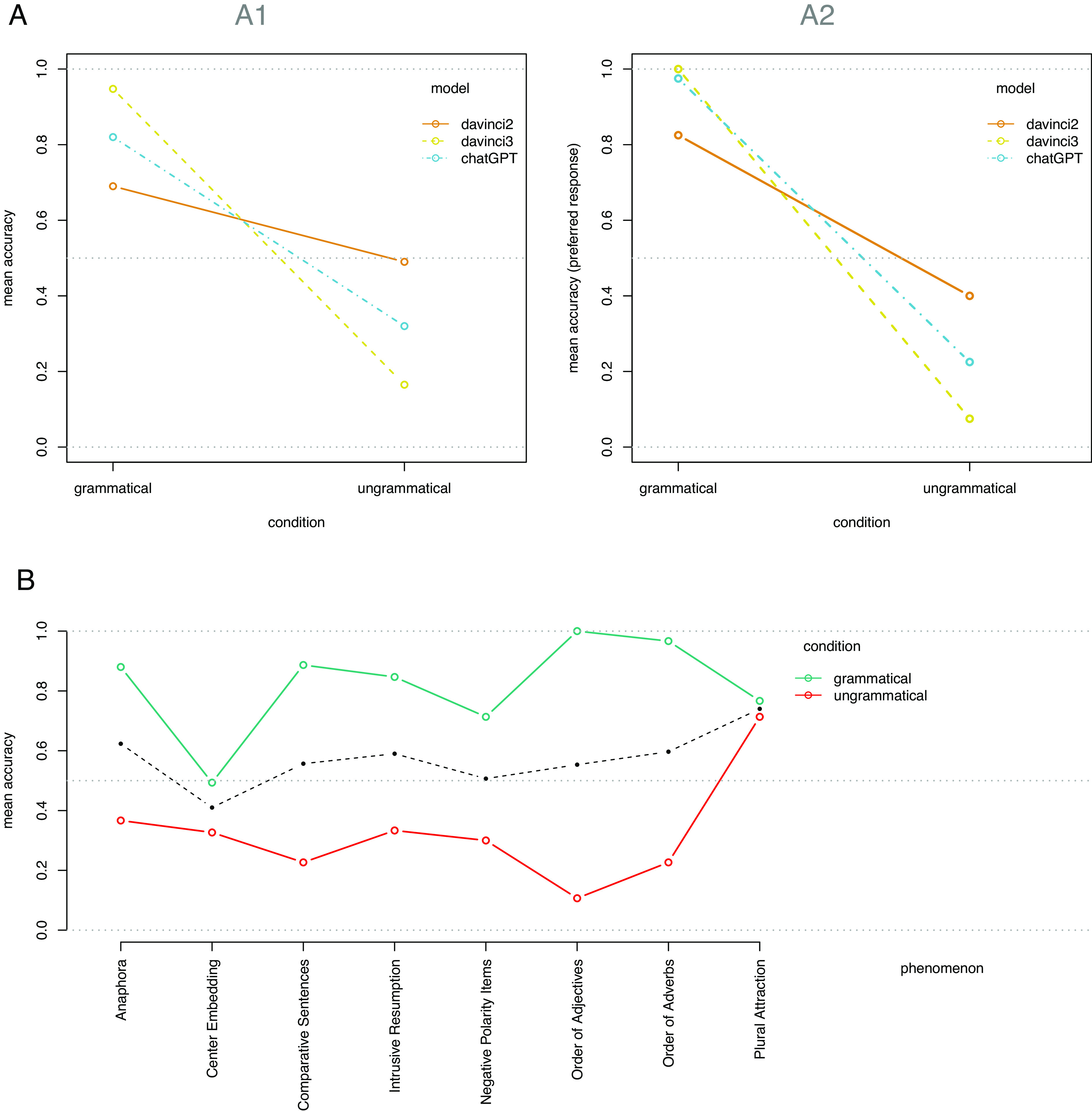
(*A*) Mean accuracy by condition and model: (*A1*) individual responses; (*A2*) preferred responses per sentence. (*B*) Mean accuracy by phenomenon and condition. The dashed black line indicates the mean accuracy for each phenomenon across both conditions.

The accuracy rates by model and condition (grammatical/ungrammatical) are shown in Fig. 1*A1*. The intercept of an intercept-only GLMM was significantly different from zero (β = 0.585, z = 2.61, *P* = 0.009), indicating that the overall LM accuracy is above chance, but at an absolute mean accuracy rate of M = 0.572, it is also clearly far from perfect. Further LR-tests indicate a significant main effect of condition (X2(1) = 68.41, *P* < 0.001). None of the three models performs above chance level for ungrammatical sentences (Fig. 1*A1*). Adding a main effect for the factor model did not improve the GLMM (LR-test: X2(2) = 2.97, *P* = 0.227), indicating that there are no significant overall differences between mean accuracies of 0.590 for davinci2, 0.556 for davinci3, and 0.570 for ChatGPT.^*^

However, a GLMM including an interaction between both factors significantly outperformed the condition-only model (X2(4) = 235.84, *P* < 0.001). Thus, models that are more accurate in the grammatical sentences are at the same time less accurate in the ungrammatical sentences, to the degree that there is no discernible overall difference between models. This demonstrates a general response bias for the yes-response rather than a genuinely higher accuracy (overall, 74.7% of responses are yes-responses; 60.0% for davinci2, 89.1% for davinci3, and 75% for ChatGPT). Thus, the almost top-level performance of davinci3 for grammatical sentences (CI.95 = [0.954, 0.987] for the model intercept when these factor levels are set as reference conditions) is offset by its very low accuracy for ungrammatical sentences (CI.95 = [0.079, 0.187]), leaving it at an overall accuracy not higher than the other two models.

##### Differences between grammatical phenomena.

In a follow-up analysis, we examined whether there are differences in model accuracy for the different grammatical phenomena we tested. Since we considered different phenomena as a general source of variation, we had no specific hypotheses as for which phenomena we expect higher or lower accuracy and therefore analyzed this variable only at the factor level.

We observed a main effect of condition (LR-test: X2(1) = 68.39, *P* < 0.001), an additional main effect of phenomenon (X2(7) = 14.18, *P* = 0.048), and an additional interaction between the two (X2(7) = 102.91, *P* < 0.001). The mean accuracy rates by phenomenon and condition are displayed in Fig. 1*B*. As can be seen, the accuracy for ungrammatical sentences is lower than for grammatical sentences across all phenomena. Plural attraction is the only phenomenon for which the performance for ungrammatical sentences is above chance level (β = 0.929, z = 3.98, *P* < 0.001 for the intercept in the GLMM with this condition as the reference level).

#### Stability.

To examine to what extent LMs provide stable responses, we analyzed two different metrics measuring two different aspects of (in)stability (Table 1): Oscillation is a local measure, defined at the individual trial level as the number of trials in which the LM judgment for a given prompt differs from the one given at the previous trial. Deviation is a global measure defined at the sentence level, that indicates how often the LM provides the less frequent response. Deviation is therefore directly and strictly monotonously related to the Shannon entropy of the response distribution, but scales in a linear manner, which is more adequate for an analysis with linear models. Examples for these two measures, and how they differ for different response sequences, are provided in Table 1.

**Table 1.** Illustration of the differences between the oscillation and deviation measures of response instability

Response sequence for sentence repetition	Oscillations (trial level)	Overall oscillations	Deviations
YYYYYYYYYN	−000000001	1	1
YYYYNYYYYY	−000110000	2	1
YYYYYNNNNN	−000010000	1	5
YNYNYNYNYN	−111111111	9	5

In the first and third rows, the same number of oscillations comes with large differences in deviation; in the third and fourth row, the same deviation comes with large differences in oscillations. Since the first occurrence of each sentence cannot differ from the previous response, it does not provide data points for the oscillation variable.

##### Stability by model and condition.

**Fig. 2.**
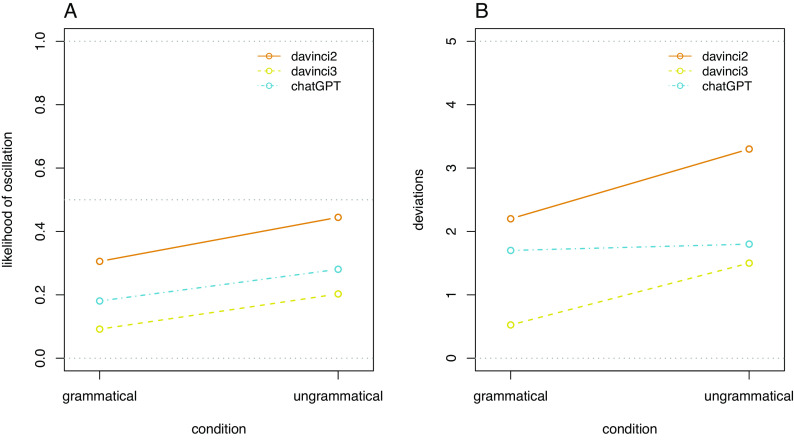
Response instability by model and condition. (*A*) Instability measured as likelihood of oscillations. (*B*) Instability measured as the number of deviations.

For oscillations, the intercept in an intercept-only GLMM is significantly below zero (β = −1.28, z = −10.91, *P* = <0.001), indicating that the probability of observing an oscillation is less than fifty percent and thus consecutive responses are not completely random. At the same time, the CI for the likelihood of an oscillation at CI.95 = [0.181, 0.259] does not include zero, indicating a considerable degree of instability in the responses. We find an additional main effect of model (LR-test: X2(2) = 114.39, *P* < 0.001), indicating a difference in stability between the three models, and an additional main effect of condition (X2(1) = 13.68, *P* < 0.001), but no significant interaction between these two factors (X2(2) = 2.60, *P* = 0.272). The effects of both factors on oscillations are displayed in Fig. 2*A*, showing the lowest number of oscillations for the davinci3 model (followed by ChatGPT and davinci2) and grammatical sentences. However, even when these most stable factor levels were set as the reference levels, the intercept of the GLMM did not include zero (CI.95 = [0.054, 0.110]), demonstrating some degree of instability even there. When doing the same for the least stable case (davinci2, ungrammatical sentences), the intercept is not significantly different from zero (β = −0.21, z = −1.24, *P* = 0.216), indicating that a response is not predictable from the previous response for the same sentence.

In the intercept-only LMM for deviations, this intercept is significantly higher than zero (b = 1.84, t(79) = 14.77, *P* = <0.001), indicating that the models provide some deviating responses across all items (1.84 on average; CI.95 = [1.59, 2.08]). Here, we find a significant main effect for model (LR-test: X2(2) = 56.77, *P* < 0.001), for condition (X2(1) = 9.10, *P* = 0.002), and for their interaction (X2(2) = 6.79, *P* = 0.033). The effects of both factors on deviation are displayed in Fig. 2*B*. Even in the most stable case (davinci3, grammatical sentences), there is a non-zero number of deviating answers (CI.95 = [0.06, 0.99]). In the most unstable case (davinci2, ungrammatical sentences), the number of deviating answers is very high but still differs from the maximum value of five (CI.95 = [2.83, 3.77]), indicating that despite the high instability, the model still tends to “prefer” one response alternative.

In the interpretation of these results, it is again important to note that a higher response stability can be caused by a general response bias toward the “yes” or “no” answers, which (considering the results of the accuracy analysis) is a very likely reason why responses by the davinci3 model are more stable.

#### The Interplay between Stability and Accuracy.

So far, we have seen that all LMs show clear deficits with respect to both accuracy and stability. However, these variables are intertwined, as stability sets clear bounds to the minimum and maximum level of accuracy possible (the number of deviations, and the number of repetitions minus the number of deviations, respectively). Here, we examine in more detail two ways in which the lack of stability could affect accuracy: Specifically, first we test whether the lack of accuracy is a direct consequence of the lack of stability. Second, we test whether the lack of stability might play out in a positive direction, allowing the model to repair initially incorrect responses over repetitions of the same prompt.

##### Does the lack of accuracy follow from the lack of stability?

Given that the last analysis has shown that all models in all conditions show some degree of preference for one of the two responses, it may be the case that we find that the models provide accurate answers once we factor out the stability factor by only considering these preferred answers for each item (i.e., discarding and ignoring all deviations).

In this GLMM analysis (with the preferred response as only one data point per sentence and model), we observe a main effect for condition (LR-test: X2(1) = 82.59, *P* < 0.001), no main effect for model (X2(2) = 2.51, *P* = 0.286), and an interaction between the two factors (X2(2) = 27.40, *P* < 0.001). The effects of both factors on preferred-response accuracy are shown in Fig. 1*A2*. As can be seen, the pattern looks very similar to the standard accuracy analysis (Fig. 1*A1*), albeit with overall higher accuracy rates for grammatical sentences and lower accuracy rates for ungrammatical sentences. Here, the top-performing combination of factors (davinci3, grammatical sentences) reaches the maximum accuracy of 100%, but this is again offset by the very low accuracy of this model for ungrammatical sentences. Therefore, the lack of accuracy reported above is not simply a consequence of the instability in responses; if anything, this instability somewhat corrects for the response bias toward yes answers (85.0% yes-responses for the preferred responses vs. 74.7% yes-responses in the trial-level data).

#### Does accuracy improve over repetitions?

**Fig. 3.**
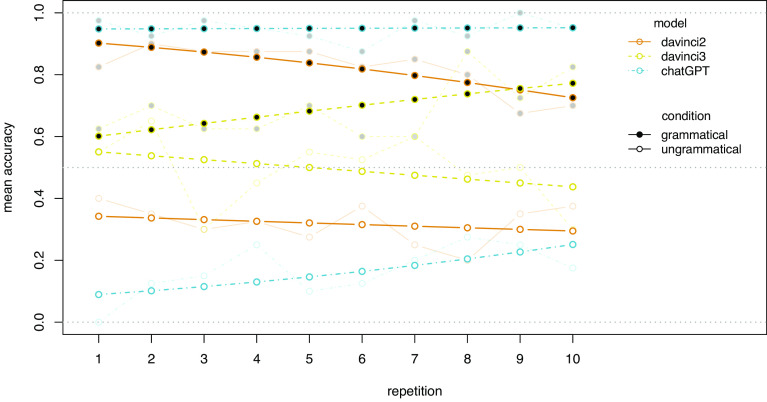
The effect of repetitions on mean accuracy by model and condition. The transparent points represent the observed data; the opaque points represent the predictions of the GLMM including a three-way interaction between the factors.

As described above, it is possible that the lack of stability actually runs in a positive direction, reflecting a repair process by which the LMs learn, over the course of multiple repetitions of the same prompt, to correct initial errors and generate more accurate responses. To test this possibility, we started from the GLMM including an interaction between model and condition reported above (Fig. 1*A1*) as a baseline. In an LR-test, we observed no additional main effect of repetitions (X2(1) = 0.00, *P* = 0.992), indicating no general positive trend for higher accuracy rates after more repetitions. We further observed an interaction between model and repetition (X2(2) = 14.02, *P* = 0.001), no interaction between condition and repetition (X2(1) = 0.04, *P* = 0.849), and a significant three-way interaction between model, condition, and repetition (X2(2) = 15.86, *P* < 0.001). The effects of repetitions by model and condition are displayed in Fig. 3. The only model-condition combination for which Fig. 3 suggests some improvement over repetitions is ChatGPT for ungrammatical sentences; the increase in accuracy for grammatical sentences by davinci3 is again offset by a simultaneous decrease of the same magnitude for ungrammatical sentences, indicating an increased general bias toward yes-responses. Interestingly, for davinci2, we observe a decrease in accuracy over repetitions over both conditions, which is more prominent for grammatical sentences.

#### Does stability improve over repetitions?

**Fig. 4.**
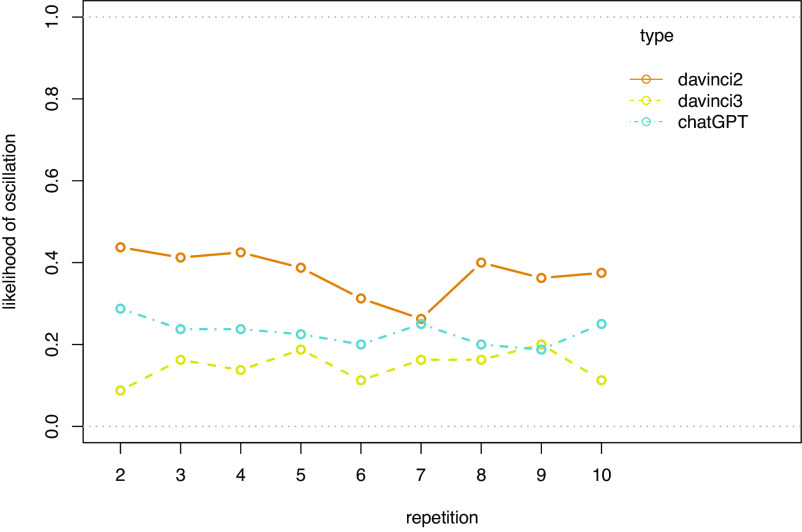
Response instability by model and repetitions, measured as the likelihood of oscillations.

While the previous analysis showed that LM accuracy does not improve over repetitions, it might be the case that they at least converge on a stable response pattern (be it accurate or inaccurate) as they repeatedly respond to the same prompt. In a further analysis of oscillations, we find that this is not the case: Adding a fixed effect of repetitions does not improve upon a GLMM already containing an interaction effect of model and condition, neither as a main effect (X2(1) = 1.33, *P* = 0.249), nor in a two-way interaction with the model (X2(3) = 3.89, *P* = 0.274) or condition (X2(2) = 1.47, *P* = 0.479), nor in a three-way interaction (between repetitions, model, and condition) (X2(6) = 4.56, *P* = 0.602). The effects of repetitions on stability are displayed in Fig. 4.

#### Comparisons with Human Data.

**Fig. 5.**
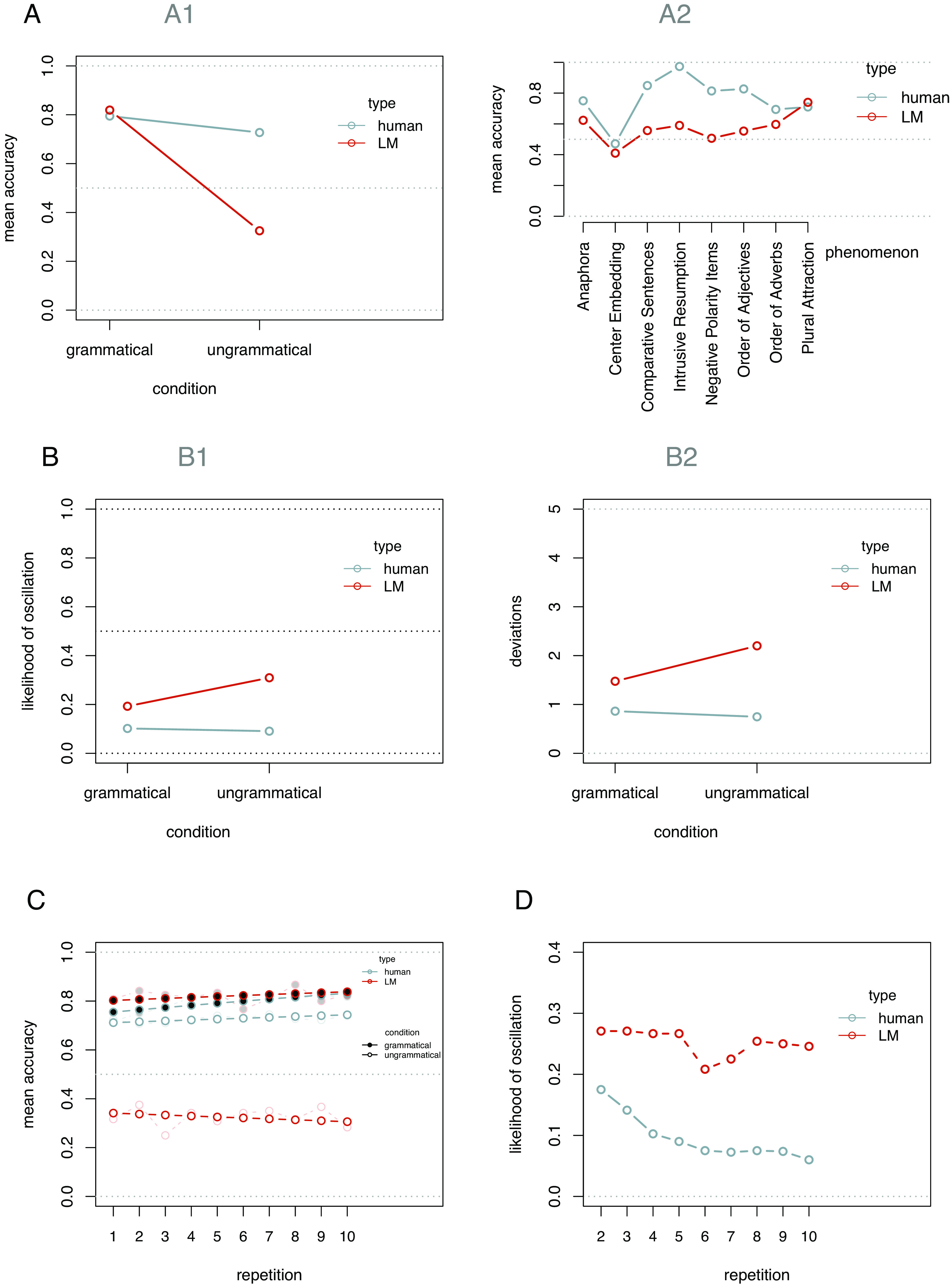
(*A*): (*A1*) Mean accuracy by type of responding agent and condition. (*A2*) Mean accuracy by type of responding agent and phenomenon. (*B*) Response instability by type of responding agent and condition: (*B1*) Instability measured as likelihood of oscillations; (*B2*) Instability measured as the number of deviations. (*C*) The impact of repetitions on accuracy by type of responding agent and condition. (*D*) The impact of repetitions on stability by type of responding agent.

Overall, our analyses showed considerable differences between humans and LMs in terms of both accuracy and stability and how these are affected by repetition (Fig. 5).

##### Accuracy.

For accuracy, the non-significant main effect of type of responding agent (i.e., humans vs. LMs) indicates no difference between the two for grammatical sentences (the reference condition; β = 0.48, z = 0.48, *P* = 0.635), while the significant interaction term (β = 1.94, z = 15.46, *P* < 0.001) indicates a substantial difference for ungrammatical sentences (Fig. 5*A1*), with humans outperforming LMs. For a more detailed depiction of the accuracy rates of humans and LMs by grammatical phenomenon, see Fig. 5*A2*.

###### Stability.

Humans exhibit a more stable response pattern than LMs (Fig. 5*B*). In these analyses, we set human participants as the reference condition for the factor type of responding agent. For oscillations, again, we find no main effect of the type of responding agent (β = 1.03, z = 1.64, *P* = 0.102), indicating no difference between humans and LMs for grammatical sentences, no main effect of condition (β = −0.12, z = −0.81, *P* = 0.417), indicating no difference between grammatical and ungrammatical sentences for humans, but a significant interaction (β = 0.84, z = 6.13, *P* = <0.001), showing more oscillations in the LM responses compared to the human responses for ungrammatical sentences (Fig. 5*B1*). The exact same pattern emerges for the deviations (b = 0.61, t(52) = 1.63, *P* = 0.109 for type; b = −0.11, t(76) = −1.15, *P* = 0.256 for condition; b = 0.84, t(858) = 4.75, *P* < 0.001 for their interaction); see Fig. 5*B2*.

###### Does accuracy improve over repetitions?

The GLMM that best describes the accuracy data includes all three two-way interactions between repetitions, type of responding agent, and condition, but not additionally their three-way interaction (LR-test: X2(1) = 2.86, *P* = 0.091). This model reveals that a) in line with the previous analysis, the LM accuracy for ungrammatical sentences decreases with repetitions (β = −0.05, z = −2.59, *P* = 0.010 for the condition*repetitions interaction), while critically b) human accuracy increases more with repetitions than LM accuracy (β = 0.05, z = 2.25, *P* = 0.024, for the type*repetition interaction) and c) again humans are far more accurate for ungrammatical sentences than LMs (β = 1.93, z = 15.40, *P* < 0.001 for the type*condition interaction). Thus, as can be seen in Fig. 5*C*, human accuracy—unlike LM accuracy—improves for both grammatical and ungrammatical sentences.

###### Does stability improve over repetitions?

In a GLMM predicting oscillations, we find that LM stability does not improve over repetitions (β = −0.02, z = −1.11, *P* = 0.265 for the repetition main effect). We also find no significant difference between LMs and humans at the first presentation of a sentence (β = −0.71, z = −1.09, *P* = 0.277 for the type main effect). However, we observe a significant interaction term (β = −0.14, z = −5.34, *P* < 0.001), indicating that—unlike LMs—humans show fewer oscillations the more often they respond to the same prompt (Fig. 5*D*).

## Discussion

The present work aims to inform the conversation on whether LMs can act as theories of natural language. We contribute to this discussion by investigating the claim that LMs possess human-like linguistic abilities that include learning and successfully applying rules of grammar. To this end, we prompt three LMs with a series of grammaticality judgment tasks, aiming at determining whether judgments are accurate (RQ1), stable (RQ2), and how these two factors interplay toward a possible convergence on stable and/or accurate answers (RQ3). The systematic testing of all three LMs revealed marginal overall above-chance accuracy and absence of response stability. In other words, the LM answers to questions tapping into the (un)grammaticality of prompts that pertain to different language phenomena are largely inaccurate (RQ1), ever-changing (RQ2), and not playing out in favor of a strategy that culminates in either more stable or more accurate answers (RQ3). All these characteristics are in stark contrast to what is observed in humans. Tested on the same tasks, humans provided judgments that are largely accurate across conditions (RQ1), stable (RQ2) and, if initially inaccurate, largely converging toward accurate responses over repetitions of the same prompt (RQ3).

This behavior is in line with what has been found in the linguistics literature: Humans provide reliable and robust judgments about their language(s), while also trying various repair strategies during grammatical processing. Of course, humans are not perfectly accurate in such judgment tasks because language variation is bound to give rise to some variability in the judgments. For example, one of our grammatical examples is the following: “The game that the child who the lawnmower had startled in the yard was playing in the morning lasted for hours.” This sentence is labeled grammatical in the dataset (following ref. 25), but many grammatical sticklers would say that “who” should be “whom”, thus judge it as wrong. This limited interspeaker variation, together with performance factors (e.g., fatigue, temporary distraction, involuntary hit of the wrong key in an experimental setting), explains why humans performed significantly better than LMs both in terms of stability and accuracy, without however showing at ceiling performance.

LMs, on the other hand, are algorithms trained on generating probability distributions over tokens in the input data for text-generation purposes. A straightforward empirical expectation would then be for LMs to be able to predict whether a group of words can, or cannot, pattern together in a language that they presumably use in a human-like manner. In this respect, we found several effects disconfirming this expectation. First, all three LMs’ mean accuracy is just above chance. The fact that the LMs fail at providing accurate judgments of well-formedness, notwithstanding the fact that they have been linked to (nearly) achieving mastery over form (21), suggests that this framing may need to be rethought. If one endows LMs with human-like competence over form, it is unclear what prevents the LMs tested in our experiment from producing target grammaticality judgments in a consistent and reliable way. Specifically, while overall accuracy is slightly above-chance, no LM performs above chance when questioned on ungrammatical prompts, as opposed to humans, whose accuracy is well above chance for both grammatical and ungrammatical sentences. The fact that the LMs largely provide correct yes-responses to questions that feature grammatical prompts but that higher accuracy in the grammatical condition invariably correlates with lower accuracy in the ungrammatical condition (where yes is the wrong response), demonstrates that the tested LMs i) are not sensitive to the violations behind ungrammatical prompts and ii) are biased toward yes-responses.

Insensitivity to (un)grammaticality amounts to a qualitative mismatch problem between LM language and human language. LMs which cannot contribute to the description of the language they have been trained on, by recognizing and ruling out its ungrammatical instances, fail to be “observationally adequate” (33): They are incapable of figuring out the limits of that language’s variation. This failure to discern possible from impossible word patterns makes LMs run into a problem of over-recognition and consequently over-generation, as the innate constraints (or lack thereof) of their architecture allow them to produce outputs that, while superficially similar to natural language, are indeed not natural language (34).

In LMs, failure to recognize ungrammaticality occurs despite the fact that the models have exposure to human feedback that provides evidence of the negative type (35). This exposure should enhance their capacity to distinguish between inputs that comply with the target language and those that deviate from it. In other words, LMs differ from humans in one important respect: the type of evidence they have access to. For LMs, reinforcement learning of this sort marks an important difference from child language acquisition because children do not have access to negative evidence. While caregivers may occasionally reformulate a child’s utterance that is deemed incorrect (36), no child systematically receives negative feedback for constructions that do not exist in their target language (37–40). More importantly, the caregiver corrections are often not contingent on grammatical correctness, but rather concern phonological and semantic errors (39). Even when adult feedback targets grammatical correctness, it typically consists of mere reformulations, without elaborating on why a sentence is ungrammatical. In other words, children—unlike LMs—learn language without being provided with rich auxiliary feedback that explains the ways an incorrect sentence deviates from the target rule of grammar (40). This means that there is an important double mismatch in the input and output of humans vs. LMs: i) LMs receive rich information about strings of words that correspond to grammatically wrong sentences, while humans do not, but still, ii) they are not able to accurately judge grammatically wrong sentences as such, while neurotypical humans can.

In our experiment, the failure of LMs to rule out ungrammatical sentences is accompanied by an inability to recognize some grammatical prompts as such, despite the strong yes-response bias. In particular, performance on the 8 tested linguistic phenomena seems to vary as a function of the ease of rendering structural relations in linear terms. In language, relations between words are defined structurally and not linearly (41). In our results, the LM inability to recognize prompts that are grammatical (i.e., structurally legitimate), but whose linear word-order makes them look like they are not, is a sign of LMs lacking competence over structural relations. For instance, the LM accuracy on grammatical prompts of center-embedded sentences is at chance, notwithstanding the strong yes-response bias found in all LMs. On the other hand, for phenomena whose structural relations can be more readily coded in terms of linear co-occurrence (e.g., in plural attraction, number agreement patterns can be defined in terms of the verb agreeing with a semantically appropriate noun), accuracy is high even on ungrammatical prompts. Taken together, this inability to spot ungrammatical prompts as falling outside the domains of what a target language actually permits, together with the failure to consistently recognize grammatical prompts as such, are an index of a performance mismatch between humans and LMs.

LMs typically produce language outputs that are largely grammatically correct, but is this enough evidence to claim that they have a human-like understanding of language? It seems there are reasons to believe that AI does not actually approximate human cognitive (including linguistic) behavior neither at the computational (42) nor at the phenotypic (5) level. While it has been argued that metalinguistic judgments do not give conclusive evidence for a model’s capacities (43), the differences we found between LMs and humans attest to foundational dissimilarities in their performance: Humans are consistent in their judgments both at the individual and at the group level, whereas the LMs fail to be accurate and, crucially, consistent even at the individual level. From a methodological point of view, if the existence of fluent and grammatically correct LM outputs is used as an argument that supports their human-like knowledge of grammar, then performance that markedly deviates from human linguistic behavior should count as counterevidence that challenges the presence of such knowledge (5).

Turning attention to the mechanisms that guide answers, all LMs show a strong bias toward yes-responses. In the literature, evidence has been found of Machine Learning algorithms magnifying biases (e.g., racial, sexist, political) present in their input, giving rise to real-world consequences (44–46). Adding to such evidence, the present experiment finds a bias that is more general in nature. Regardless of the fact that LMs might be programmed as non-deterministic systems, such that the generation of the same answer to a given prompt seems dispreferred, a basic requirement for LMs to be reliable would be for this non-determinism to only affect the paraphrasing of content, rather than its truth value. In the grammaticality judgment tasks we employed, the choice between yes or no as answers to the question of whether the tested sentences are grammatically correct straightforwardly affects truth values (as yes and no contradict each other, and no sentence can be both grammatical and ungrammatical at the same time). The fact that the three LMs we tested systematically favor one answer over the other, irrespective of task conditions (grammatical vs ungrammatical), renders claims about the LM ability to encode meaning hard to sustain (22, 47, 48).

The presence of such a bias in the LM responses could be attributed to engineered preferences that make use of default strategies for answering. As mentioned above, the models are non-deterministic and lack a grasp of truth. Their aim is to provide a correct answer as often as possible, and to maximize the probability of providing correct answers, it is possible that they recruit different built-in strategies for answering: from defaulting to a standard yes response when the answer to a prompt is unclear, to switching responses when the same question is prompted repeatedly, utterly disregarding clashing truth values. Another possibility is that this bias relates to frequencies in the training data. It has been shown that the accuracy of LMs in question-answering is directly linked to the count of documents containing answers for the question being asked (49) and that, more generally, the performance of LMs on reasoning tasks correlates with the frequency of relevant terms in the training data (50). Although no specific information is available about the texts on which the tested LMs have been trained, it seems certain that the training materials in grammatical English vastly outnumber those failing to comply with basic rules of grammar. Both explanations we offer for the observed bias are mere speculations; what causes the LMs to accept as grammatical certain sequences of tokens that are not part of the tested language is a result left with no firm explanation. If, however, one reconsiders the basic premise that ascribes human-like language abilities to LMs, our results are more straightforwardly explained: The tested models are able to answer the questions we posited, but they have learned neither the relevant rules of grammar that govern the use of words nor their meaning.

The third finding of our experiment concerns the instability of responses. Had stability measures revealed a high rate of agreement in judgments concerning the exact same prompt, this would have allowed the inference that some sort of grammatical processing strategy (however wrong or correct) was employed by the LMs in order to navigate the task demands. The absence of decision stability forces us instead to contemplate absence of deployment of either task-appropriate or -inappropriate processing heuristics and/or rules [cf. (51)]. Observing all three LMs repeatedly provide different answers to the same question is in stark contrast to what we saw for humans: While humans provided judgments that are occasionally inaccurate for reasons that relate to performance factors and cognitive effort thresholds (13, 52, 53), they were nonetheless largely consistent and stable in their replies [cf. (11)]. Moreover, humans showed increasing stability of answers as the number of repetitions of a given prompt increased, again in contrast to what LMs did. Therefore, independently of accuracy, the absence of stability in LMs further complicates determining what exactly drives their performance. The absence of a directionality pattern in the responses (i.e., the tendency to proceed from non-target to target judgments, or vice versa) can be framed under a similar light, namely, as revealing a strategy void that largely leaves performance to chance, engineered preferences aside.

One potential challenge to our results goes through arguing that the tested LMs showed low language accuracy and high response instability because either they have not understood the task at hand, or they are not able to perform such a task in the first place. With respect to the latter, when a prompt is not parsed due to some temporary glitch (that may or may not be related to the nature of the prompt), the application interface issues error warnings. For instance, if ChatGPT is asked to upload a YouTube video of itself or reveal the secret of immortality, it may issue warnings that suggest that either the request was not completed successfully because an unexpected error occurred, or that it is an AI not capable of performing the request. In other words, when LMs cannot complete a request successfully, they are programmed to warn the user and acknowledge such limitations. In our testing sessions, no such error warnings occurred. In all likelihood, this suggests that LMs are indeed able of handling prompts that ask them simple questions about the one thing that is abundant in their training data: language. With respect to the possibility that LMs completed the tasks we administered without, however, truly understanding what the question “Is sentence X grammatically correct in English?” means, two issues merit unpacking.

First, if by “not understanding” one means “not understanding in a human-like way”, this reading is in line with our results that challenge the popular claim that LMs have human-like language abilities. Concisely, we do not ascribe a human-like language system to LMs; in fact, this is precisely what we aimed to put to test, and the results we obtained challenge the conclusion that LMs have human-like language abilities.

Second, taking issue with the assumption that LMs should be able to perform simple grammaticality judgment tasks—while claims about LMs showing human-like language understanding, sentience, and an ability to extrapolate morphophonological rules of natural language in a way comparable to language learning by humans (54, 55) abound—, shifts the burden of proof from proponents to critics. This is an undesirable move because it makes the initial proposition (i.e., LMs have human-like language abilities) empirically unfalsifiable, as any test that works for humans may be challenged as unsuitable and not of the right granularity for LMs.

To conclude, our experiment shows that the tested LMs display insensitivity to possible vs. impossible language, with their answers being both largely inaccurate and guided by a yes-response bias. The reason behind this failure to distinguish grammatical from ungrammatical sentences can be traced back to Chomsky’s view that the definition of grammaticality cannot depend on a) a sentence being in a corpus, b) a sentence being meaningful, and c) the probability of it being uttered (56). Since AI models are precisely using a) and c), they are unable to master the distinction in question. While not taking issue with the potential value of LMs as tools for certain tasks, the results of the present work challenge the claim that LMs possess human-like language abilities. Such results are ultimately incompatible with the view that the tested LMs can serve as cognitive models of the human language capacity at their current stage of development.

## Supplementary Material

Appendix 01 (PDF)

## Data Availability

XLSX, CSV, R, DOCX, PDF, TXT data have been deposited in the OSF (https://osf.io/7ajxr/) (57).

## Footnotes

^*^An analysis of the d′ measure that considers both the hit rate and the false alarm rate in a single measure replicates the same pattern of a slightly above chance performance, with no difference between the three models. Specifically, we compared the models (8 phenomena × 3 models, giving a total of 24 data points), using a standard linear regression model with d′ as the dependent variable and model as a fixed effect predictor. The obtained intercept of 0.63 indicates a sensitivity slightly above chance level (which would be a d′ of zero), in line with the 0.585 overall accuracy rate reported in the original analysis.
