# Supplementary material for: Systematic testing of three Language Models reveals low language accuracy, absence of response stability, and a yes-response bias
Source: Proc Natl Acad Sci U S A. 2023 Dec 13;120(51):e2309583120. doi: 10.1073/pnas.2309583120 (PMC10743380; doi:10.1073/pnas.2309583120)
Supplement: Supplementary file 1 — Appendix 01 (PDF) [file pnas.2309583120.sapp.pdf]

PNAS

Supporting Information for

Systematic testing of 3 Language Models reveals low language accuracy, absence of response stability, and a yes-response bias

Vittoria Dentella[1]*, Fritz Günther[2] and Evelina Leivada[3,4]

[1] Universitat Rovira i Virgili, Tarragona, Spain
[2] Humboldt-Universität zu Berlin, Berlin, Germany
[3] Universitat Autònoma de Barcelona, Barcelona, Spain
[4] Institució Catalana de Recerca i Estudis Avançats (ICREA)

*Vittoria Dentella
Email: vittoria.dentella@urv.cat

This PDF file includes:

Table S1

Table S1. Summary of the tested phenomena.

**PLURAL ATTRACTION**
**Grammatical: singular subject, plural attractor, singular verb**
a. The key to the cabinets probably was destroyed by the fire
b. The picture on the fliers definitely was of a village church in the south of France
c. The label on the containers probably was a warning about the hazardous chemicals inside
d. The crime in the suburbs doubtlessly was a reflection of the violence in today's society
e. The entrance to the exhibits evidently was hard to locate on the diagram
**Ungrammatical: singular subject, plural attractor, plural verb**
f. *The slogan on the posters unsurprisingly were designed to get attention
g. *The mistake in the programs certainly were disastrous for the small software company
h. *The problem in the stores ultimately were solved by firing the custodian
i. *The defect in the appliances likely were unknown to consumers and government regulators
j. *The door to the laboratories accidentally were left unlocked by the cleaning service
**ANAPHORA**
**Grammatical: anaphoric match**
The young caddy who escorted the experienced golfers supposedly dirtied himself by falling in the sand
**Ungrammatical: anaphoric mismatch**
*The grimy blacksmith who worked for the royal knights evidently hurt themselves with the sharp tools
**CENTER EMBEDDING**
**Grammatical: 3 verb phrases**
The ancient manuscript that the grad student who the new card catalog had confused a great deal was studying in the library was missing a page
**Ungrammatical: 2nd verb phrase missing**
*The trophy that the athlete who the restaurant had hired as a spokesman was stolen later
**COMPARATIVE SENTENCES**
**Grammatical: plural embedded subject**
More gym instructors won a marathon yesterday than lawyers did
**Ungrammatical: singular embedded subject**
*More photographers won their third Pulitzer this year than the professor did
**INTRUSIVE RESUMPTION**
**Grammatical: gap**
This is the boy that the cop who was leading the operation beat up
**Ungrammatical: intrusive resumptive pronoun**
*This is the mug that the artisans who were working with ceramics crafted it
**NEGATIVE POLARITY ITEMS**
**Grammatical: negation licensor**
No authors that the critics recommended have received any acknowledgment for a best-selling novel
**Ungrammatical: irrelevant negation licensor**
*The professors that no students respected have ever wanted negativity in a class debate
**ORDER OF ADJECTIVES**
**Grammatical: *pro* hierarchy**
I bought a nice small German electric bike
**Ungrammatical: *contra* hierarchy**
#I saw a vitreous Venetian tall graceful vase
**ORDER OF ADVERBS**
**Grammatical: *pro* hierarchy**
Melissa then always cleaned the dishes
**Ungrammatical: *contra* hierarchy**
#Sonja already probably set the table
